# Scrotal granulomatous aspergillosis in a dromedary camel (*Camelus dromedarius*)

**DOI:** 10.1186/s12917-017-1001-z

**Published:** 2017-03-29

**Authors:** Frine Eleonora Scaglione, Andrea Peano, Sara Piga, Stefano Meda, Enrico Bollo, Francesca Tiziana Cannizzo, Mario Pasquetti, Henrik Elvang Jensen

**Affiliations:** 1 Dipartimento di Scienze Veterinarie, Università degli Studi di Torino, Largo Paolo Braccini 2, 10095 Grugliasco (To), Italy; 2 Zoom Torino, Strada Piscina 36, 10040 Cumiana, Italy; 3 Department of Veterinary Disease Biology, University of Copenhagen, Ridebanevej 3, DK-1870 Frederiksberg C, Copenhagen Denmark

**Keywords:** Dromedary, Camelids, Immunohistochemistry, *Aspergillus*, Scrotum

## Abstract

**Background:**

This report describes a case of primary subcutaneous aspergillosis in a 7-year-old neutered male dromedary camel (*Camelus dromedarius*).

**Case presentation:**

The animal developed a large nodular lesion in the right scrotum two years after surgical intervention for neutering. The mass had a firm consistency and was painful at palpation. Histopathology revealed dermal granulomatous inflammation with a necrotic centre, surrounded by plasma cells, macrophages, neutrophils, and sparse fungal hyphae characterised by parallel cell walls, distinct septa, and dichotomous branching. Fungal culture was not performed, but a panel of mono- and polyclonal antibodies specific for different fungal genera identified the hyphae as *Aspergillus* sp.

**Conclusions:**

The occurrence of subcutaneous lesions is a rare manifestation of aspergillosis in animals, and this appears to be the first case reported in the dromedary camel.

## Background

*Aspergillus* species are ubiquitous environmental fungal organisms [1]. They have been found worldwide in humans, in almost all domestic animals, birds as well as in many wildlife species, causing a wide range of diseases from localized infections to fatal disseminated forms [2]. Invasive aspergillosis in humans is typically associated with pulmonary infection in immunosuppressed patients [3]. However, a number of extrapulmonary localizations has been reported, sometimes in immunocompetent individuals [1]. Cutaneous aspergillosis occurs relatively less frequently, as either primary or secondary infection [3, 4]. Primary cutaneous aspergillosis usually involves sites of skin injury of various nature, while secondary lesions result either from contiguous extension to the skin from infected underlying structures or from widespread blood-borne infections [3].

In the same way aspergillosis in animals is mainly a disease of the respiratory tract (nasal cavities and lungs in mammals; trachea, lungs and air sacs in birds), although other localizations typical of particular hosts have been recognized (e.g. the guttural pouches and the cornea in horses; the retro-orbital space in cats; the intervertebral disk in dogs) [2]. Cases with cutaneous involvement are hardly found in the literature [2]. The present report describes a case of primary subcutaneous aspergillosis in a dromedary camel (*Camelus dromedarius*).

## Case presentation

**Fig. 1:**
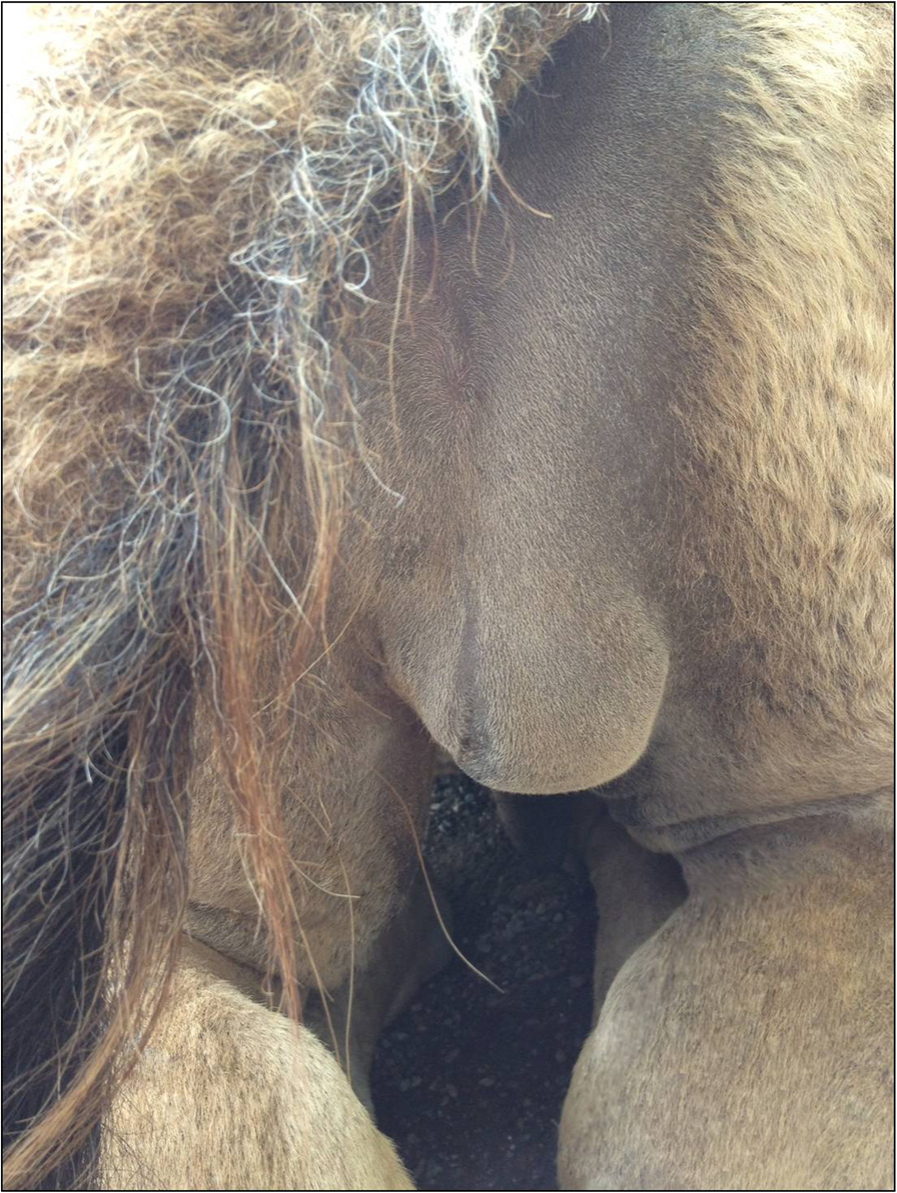
Clinico-pathological evidence of the mass in the scrotum of a dromedary camel (*Camelus dromedarius*). Subcutaneous nodular mass in the scrotum of a dromedary camel (*Camelus dromedarius*)

**Fig. 2:**
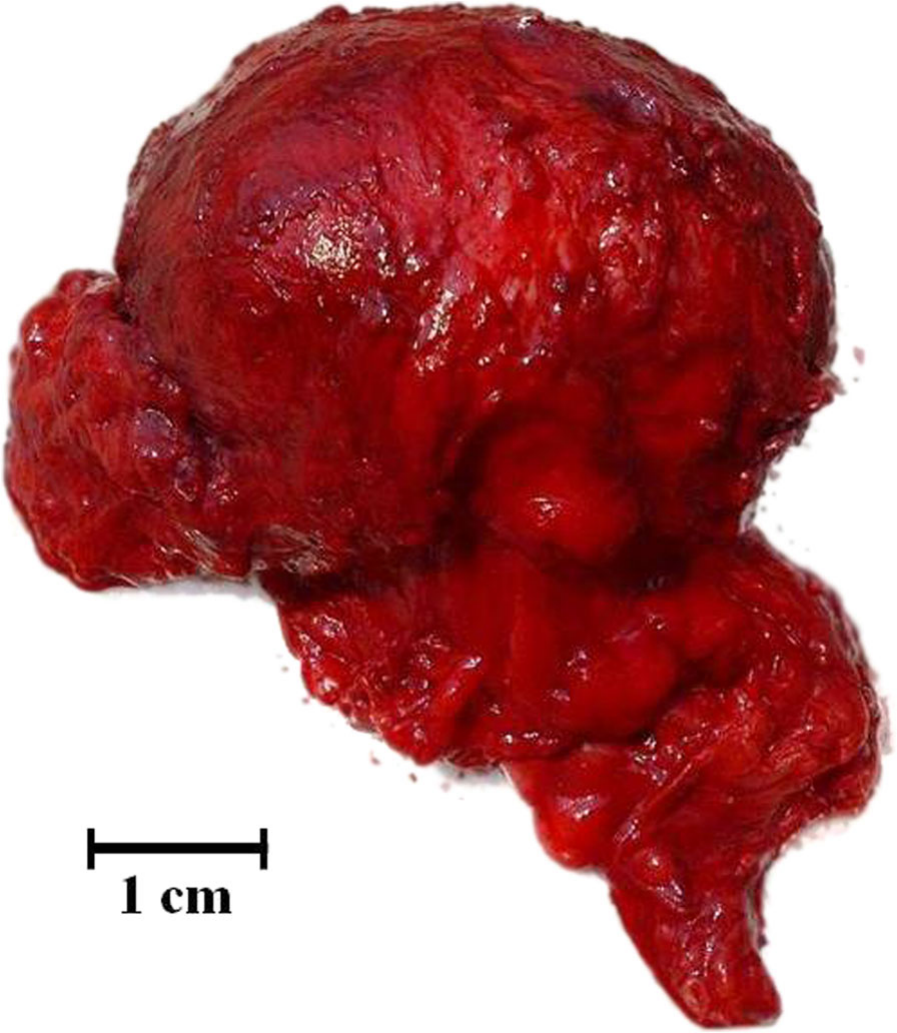
Macroscopical appearance of the mass. Encapsulated mass removed out of the scrotum of a dromedary camel

A captive 7-year-old neutered male dromedary camel, living in a zoological garden in northern Italy (Zoom Torino) and sharing the habitat with two conspecifics (a male and a female), developed a subcutaneous nodular mass in the right scrotum. The animal had been neutered two years before. A blood sample was taken from the left jugular vein and a full blood screening was performed. Differential diagnoses taken in account included an abscess and a neoplastic process. The animal was treated with antibiotics (Enrofloxacin2,5 mg/kg) given orally for 2 weeks and with anti-inflammatory drugs (Meloxicam 0,4 mg/kggiven orally for 7 days. After two months, the mass had increased (about 10 cm in diameter) (Fig. 1). At that time, it was decided to proceed with surgery. The animal was anaesthetized with a xylazine-ketamine combination and the surgical area was scrubbed with chlorhexidine solution. Both the mass and the capsule were removed. The scrotum was flushed with sterile saline solution and a drainage was placed to prevent fluid accumulation. The drainage was removed after four days, and after seven days the antibiotic therapy was discontinued. The mass (Fig. 2) was fixed in 10% neutral buffered formalin and referred to the Department of Veterinary Sciences of the University of Turin (Italy) for histological and histochemical examination. The mass was paraffin-embedded and sections of 4 μm were stained with hematoxylin and eosin.

Because fungal cultures were not performed at the time of surgery, an immunohistochemical study was carried out in order to identify of the organism. Sections of the mass were mounted on adhesive slides and kept at +4 °C until processed. Sections were examined with a panel of mono- and polyspecific anti-fungal reagents applied in various immunohistochemical techniques. In the techniques, primary reagents for immunostaining included genus-specific monoclonal and polyclonal antibodies reacting with species of *Aspergillus*, Mucorales, *Candida*, *Geotrichum, Fusarium* and *Scedosporium*, respectively [5–7]. The detecting system PowerVision + (Part No. DPVB +110AEC; Immuno Vision Technologies Co. USA) was used for signal-amplification and applied according to the manufacturer’s instructions. Sections were counterstained in Meyer’s hematoxylin for 10 s, and washed for 1 min in tap water and 4 min in distilled water. Finally, sections were mounted with glycerol-gelatin. To ensure specific reactivity of antibodies, sections were run in parallel with sections from laboratory animals experimentally infected with homologous and heterologous fungi. Moreover, in all series of staining, negative controls were run without primary reagents and with substitution of the primary antiserum or monoclonal antibody of identical isotypes, respectively, raised towards nonsense antigens.

## Results

**Fig. 3:**
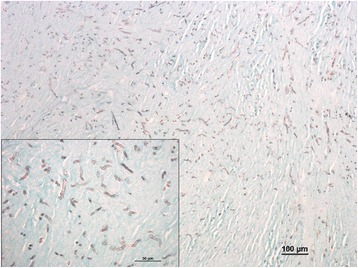
Histopathology of the mass (Grocott). Histopathology of a scrotal mass removed from a dromedary camel. Fungal hyphae characterized by parallel cell walls, distinct septa, and dichotomous branching (Grocott staining)

**Fig. 4:**
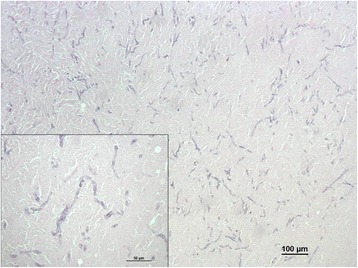
Histopathology of the mass (HE). Histopathology of a scrotal mass removed from a dromedary camel. Fungal hyphae characterized by parallel cell walls, distinct septa, and dichotomous branching (HE staining)

**Fig. 5:**
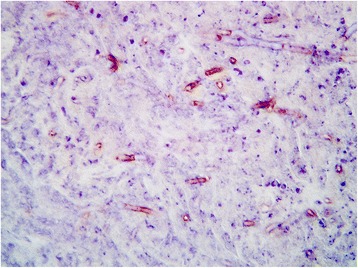
Immunohistochemical features of the mass. Specific immunolabelling of *Aspergillus* sp. in the lesion from a dromedary camel

At palpation the mass had a firm consistency and was painful. It measured 4 cm in diameter and was firmly adherent to the inner surface of the scrotum. Haematology indicated leukocytosis suggestive of a chronic inflammation, while no biochemical abnormality was detected. At the opening of the scrotum through a vertical incision, the mass appeared to be surrounded by a capsule and showed a solid appearance at the cut surface. Histopathology revealed dermal granulomatous inflammation with a necrotic centre, surrounded by plasma cells, macrophages, neutrophils, and sparse fungal hyphae. The mass was surrounded by a thick fibrous layer with multifocal lymphocytic infiltrate and haemorrhages. In order to improve the visualization of the fungal elements, periodic acid-Schiff and Grocott stains were applied to selected sections of the mass. Hyphae were characterised by parallel cell walls, distinct septa, and a dichotomous branching pattern (Figs. 3 and 4). Fungal hyphae were present only in the encapsulated granulomatous mass and no invasion of the epidermis and adjacent structures has been observed. Within the nodule removed from the dromedary the fungal hyphae only stained positive (Fig. 5) with the monoclonal antibody (clone WF-AF-1, LSBio) reacting specifically with *Aspergillus* [7, 8]. At follow up, one year after the excision of the mass, the animal was in good health with no sign of recurrence of infection.

## Discussion

This case of aspergillosis appears to be the first with a subcutaneous localization ever reported in the dromedary camel, and represents a rarity with regards to aspergillosis in animals [2]. In the dromedary, *Aspergillus* has been found associated to post-traumatic buccal infections and osteomyelitis in consequence of mandibular fractures [9] and to a clinical syndrome with multi-organic involvement, skin excluded [10]. Cases of pulmonary aspergillosis have also been reported in other camelids, such as alpaca [11], and lama [12].

Given the localization of the mass, the present case had likely a traumatic origin, represented by the surgical procedure employed to neuter the animal. The subcutaneous tissues exposed during surgery were probably colonized by spores of *Aspergillus* that induced a slow granulomatous reaction. The granulomatous inflammation with abundant fibrosis is a typical finding of chronic aspergillosis [13]. Reports in human medicine frequently describe a more rapid development of different lesions, represented by erythematous to violaceous papules or plaques that may progress to form necrotic ulcers with a central eschar, pustules and subcutaneous abscesses [14, 15]. However, also infections characterized by subcutaneous chronically slow-growing nodules have been reported [3]. In the present case it is somewhat surprising that two years after surgery the nodular mass grew to only 4 cm diameter and then a further 6 cm diameter in just two months. However, cases with a similar trend have been already described in human medicine. For example Cheetham [16] reported a patient with a subcutaneous infection by *Aspergillus* attributed to the inoculation of fungal spores occurred about one and a half year before during a course of penicillin injections. The authors speculated that the fungus lay dormant, or only developed slowly, during the year before lesion was first noted, and that the more rapid development of the mass in the later stages may have been due to the appearance of a mutant more adapted to growth in the body than the original strain [16]. Even more extreme is the case described by Lakhanpal et al. [14], regarding a 30-year-old male who developed a subcutaneous nodule due to *Aspergillus* infection over a period of 15 years.

As noted above, cases of aspergillosis with cutaneous involvement are rarely found in veterinary literature. In cats, the occurrence of skin nodules has been reported as the result of contiguous extension to the skin from infected underlying structures (nasal cavity and retro-orbital space) [2, 17]. In other cases, mostly regarding birds, the suspected pathogenesis was more similar to that of our case, i.e. on traumatic base. For example, Abrams et al. [18] described a case of *Aspergillus* blepharitis and dermatitis in a Peregrine Falcon-Gyrfalcon Hybrid (*Falco peregrinus* x *Falco rusticolus*) likely due to a trauma occurred during hunting. Copetti et al. [13] reported an outbreak of aspergillosis in some Pekin mallards (*Anas platyrhynchos*), with the involvement of different organs including the skin. The skin presented multiple elevated, yellowish brown, crusted, multifocal lesions located at the base of the feather follicles in the breast. The infection was attributed to skin trauma incited by the poultry litter [13]. Chute et al. [19] described a systemic fatal infection by *Aspergillus fumigatus* in White Rock cockerels, that was attributed to the contamination of the cutaneous incision performed to caponize the animals. Finally, in a case of some chickens, cutaneous aspergillosis was seen with generalized, tumor-like swellings, the infection being limited to the skin [20].

Primary “traumatic” aspergillosis is a well characterized condition, although rare if compared with pulmonary and systemic infections, in human medicine [3, 4]. Lesions usually develop in cutaneous sites traumatized in course of accidents or burns, but also due to surgery or minor medical procedures (positioning of intravenous catheter, drug injections, use of occlusive dressings and tapes, etc.) [3]. Most of the cases occur in patients with deficiencies in their immunological status of various reasons (AIDS, corticosteroids use, chemotherapy and immunosuppressive therapy used in organ transplants, diabetes, neonatal period and hematological disorders) [21], although primary cutaneous aspergillosis has been reported also in immunocompetent individuals [15].

## Conclusions

Although culture isolation remains the gold standard for the identification of fungi involved in human and animal infections [11], many cases are not cultured because a fungal etiology is not suspected when biological samples are sent to the laboratory [5, 6]. Even when submitted, cultures often fail to isolate the fungus responsible of the infection [8]. Despite the lack of culture in the present case, a definitive diagnosis of aspergillosis was upheld by the application of specific immunohistochemical staining techniques.
